# Medical genomics at the Systems Biology and Bioinformatics (SBB-2019) school

**DOI:** 10.1186/s12920-020-00786-x

**Published:** 2020-09-18

**Authors:** Yuriy L. Orlov, Elena N. Voropaeva, Ming Chen, Ancha V. Baranova

**Affiliations:** 1 The Digital Health Institute, I.M.Sechenov First Moscow State Medical University (Sechenov University), 119991 Moscow, Russia; 2 Novosibirsk State University, 630090 Novosibirsk, Russia; 3 Research Institute of Internal and Preventive Medicine - Branch of the Institute of Cytology and Genetics SB RAS, 630089 Novosibirsk, Russia; 4 Department of Bioinformatics, College of Life Sciences, First Affiliated Hospital of Medical School, Zhejiang University, Hangzhou, 310058 China; 5 George Mason University, Fairfax, VA 22030 USA; 6 Research Centre for Medical Genetics, 115522 Moscow, Russia

Next-Generation Sequencing-driven analysis and Systems Biology approaches commonly serve as a backdrop for a study of a tumor genome. This issue of BMC Medical Genomics SBB-2019 (“Systems Biology and Bioinformatics”) presents recent works discussed at the 11th Young Scientists School “Systems Biology and Bioinformatics”-2019, held in Novosibirsk, Russia (http://conf.bionet.nsc.ru/sbb2019/en/). Here we collated some cancer gene expression studies, some mutation profiling studies as well as some insightful case reports. The SBB school series on bioinformatics proceeds annually since 2008 under the joint steerage of the Institute of Cytology and Genetics of the Siberian Branch of the Russian Academy of Sciences and Novosibirsk State University [1, 2]. We had publications in special topic issues after the Schools before in BMC Genomics, BMC Medical Genomics and related BioMed Central family journals since 2014 [3–5]. The SBB Schools in Novosibirsk were initially conceived as satellite event for young scientists held at the same time as BGRS\SB (Bioinformatics of Genome Regulation and Structure \ Systems Biology) conference series, since 1998 taking place biannually. The recent BGRS\SB-2020 event in Novosibirsk was over at the time of the current journal issue publication (https://bgrssb.icgbio.ru/2020/). Other special issues (Supplements) to the BMC journals in the fields of genomics, genetics, bioinformatics, and medical genetics are published at BMC Genomics, BMC Genetics and three other BMC journals. The BGRS\SB-2018 conference highlights were published in 2018 [5–7], and continued the BMC Medical Genomics special issues in 2019 [8]. Public discussion of the conference presentations at the open access platforms of BioMed Central and other publishers serve as an international educational resource for young scientists [9, 10].

The articles comprising this issue of BMC Medical Genomics are focused on cancer genomics. Using transcriptomic data, bioinformatic models can be built for patient-oriented ranking of cancer drugs [11]. Nicolas Borisov et al. [12] (this issue) developed the database for cancer gene expression profiles associated with clinical outcomes of the chemotherapy treatments. Authors mined Gene Expression Omnibus (GEO), The Cancer Genome Atlas (TCGA) and Tumor Alterations Relevant for GEnomics-driven Therapy (TARGET) repositories to pull a database of 2786 gene expression profiles associated with clinical responses on chemotherapy. The cases represented breast cancer, lung cancer, low-grade glioma, endothelial carcinoma, multiple myeloma, adult leukemia, pediatric leukemia and kidney tumors and suitable for Machine Learning analysis of these malignancy.

Alexander Lavrov and co-authors [13] (this issue) review pathogenic variants targetable by single base editing. Single nucleotide variants account for approximately 90% of all known pathogenic variants responsible for human diseases, including thousands of known 6000 monogenic diseases. Recently discovered CRISPR/Cas9 base editors are capable of correcting individual nucleotide positions, thus, providing opportunities for personalized therapy. Unfortunately, none of such editors are perfect in their specificity [14]. Authors summarized all possible pathogenic variants which may be efficiently targeted by each of the known base editors. They analyzed 21 editing system currently reported in 9 publications and showed that C > T base editors are capable of precisely targeting about 3200 mutations, a total of 46% of all pathogenic T > C variants, while A > G editors may precisely target 6900 mutations (34% of all pathogenic G > A variants). Thus, even now the list of mutations which can be targeted with currently available systems is very large, and enough to choose from and embrak on developing new targeted therapies.

Next few papers highlight genomic studies of particular tumors. Anna Kudryavtseva et al. [15] (this issue) discuss mutation profiles of vagal paragangliomas, a group of rare head and neck neuroendocrine tumors, arising from the vagus nerve, and differing from more common carotid paragangliomas dissected by same group of authors earlier [16, 17]. Authors collected vagal paragangliomas from 8 patients, analyzed tumor exomes and discussed their findings in details. In particular, a number of novel and known pathogenic/likely pathogenic variants of the SDHx genes, frequently mutated in paragangliomas/pheochromocytomas were described.

Elena Pudova and colleagues [18] (this issue) analyzed miRNAs expression signatures associated with lymphatic dissemination of the locally advanced prostate tumors. Making an informed decision on PC treatment options after radical prostatectomy (with expanded pelvic lymphadenectomy) is far from being easy and depends on the stratification of patients into risk groups according to tumor stage, Gleason index, PSA level, and regional metastasis. These clinical indicators are clearly in the need of augmenting with some molecular biomarkers. The changes in miRNA expression profiles associated with lymphatic dissemination of the prostate cancer yielded a couple of miRNAs suitable for the development into the prognostic tools. The most prominent condidates, namely, miR-20a-5p, miR-106a-5p, miR-93-5p, and miR-15b-5p are well-known players in oncogenic transformation or tumor suppression [19].

Tatyana Vasilyeva et al. [20] (this issue) present a case study of congenital aniridia caused by pericentric inversion in the chromosome 11. Aniridia is a Mendelian autosomal dominant developmental disorder that can affect all eye structures as well as central nervous system, the endocrine system, and other systems and organs. In the case described, a near-megabase deletion removed a locus with *ELP4*, *PAX6*, and *RCN1* genes while the coding sequence of the *WT1* gene was not affected. The authors conclude that the risk of developing Wilms’ tumor in a probed is similar to that in the general population.

We conclude this special journal issue by the multiple paraganglioma cases report compiled by Vladislav Pavlov et al. [21] (this issue). The authors report a case of multiple paragangliomas, manifesting as bilateral carotid and vagal paragangliomas. After immunostaining for succinate dehydrogenase (SDH) subunits and exome analysis, a likely pathogenic variant in the SDHD gene was found in the germline, with additional likely pathogenic somatic variants founds in some of the tumors. It seems that authors sussessfully pinpointed germline variant in the SDHD gene as a driver of the development of multiple paragangliomas.

Overall, this issue includes reports of recent medical genomics applications in cancer and databases development, as well as case reports, continuing series on BMC Med Genomics special post-conference journal issues [10, 17, 22, 23]. We hope for continuing international exchange and education via the schools and competitions for young scientists. We invite our readers worldwide to attend the systems biology meetings in Russia. Digital Medicine Forum (Digital Medicine Forum) and MGNGS-2020 (Medical Genetics - Next-Generation Sequencing) event postponed to 2021 (http://ngs.med-gen.ru/mgngs20/).
